# Spectrum of biopsy proven renal diseases in Central China: a 10-year retrospective study based on 34,630 cases

**DOI:** 10.1038/s41598-020-67910-w

**Published:** 2020-07-03

**Authors:** Ruimin Hu, Songxia Quan, Yingzi Wang, Yali Zhou, Ying Zhang, Lu Liu, Xin J. Zhou, Guolan Xing

**Affiliations:** 1 Department of Nephrology, The First Affiliated Hospital of Zhengzhou University, Zhengzhou, Henan People’s Republic of China; 2 Department of Pathology, Baylor University Medical Center at Dallas, Dallas, TX USA

**Keywords:** Medical research, Nephrology

## Abstract

Chronic kidney diseases have become a major issue worldwide. The spectrum of biopsy proven renal diseases differs between locations and changes over time. It is therefore essential to describe the local epidemiological trends and the prevalence of renal biopsy in various regions to shine new light on the pathogenesis of various renal diseases and provide a basis for further hypothesis-driven research. We retrospectively analyzed 34,630 hospitalized patients undergoing native renal biopsy between January 1, 2009 and December 31, 2018. Indications for renal biopsy and histological diagnosis were analyzed to describe the prevalence of renal biopsy, and changing prevalence between period 1 (2009–2013) and period 2 (2014–2018) were further analyzed. Nephrotic syndrome (NS) was the most common indication for biopsy. Membranous nephropathy (MN, 24.96%) and IgA nephropathy (IgAN, 24.09%) were the most common primary glomerulonephritis (PGN). MN was most common in adults, with IgAN more prevalent in children. Lupus nephritis (LN) was the most common secondary glomerulonephritis (SGN) in adults, while Henöch–Schönlein purpura nephritis (HSPN) in children. The prevalence of MN increased significantly and nearly doubled from period 1 (15.98%) to period 2 (30.81%) (*P* = 0.0004). The same trend appeared with membranoproliferative glomerulonephritis (MPGN), diabetic nephropathy (DN) and obesity-related glomerulopathy (ORG), while the frequencies of minimal change disease (MCD), focal segmental glomerulosclerosis (FSGS), LN and hepatitis B associated glomerulonephritis (HBV-GN) significantly decreased between the two intervals. NS was the most common indication for biopsy across all age groups and genders. MN has overtaken IgAN to become the most common PGN in adults, while IgAN was the most common PGN in children. LN was the most common SGN in adults, and HSPN the most common in children.

## Introduction

Kidney disease represents one of the most important noncommunicable diseases worldwide. Approximately 10% of the world population suffers from chronic kidney disease (CKD)^1^. Similarly, the prevalence of CKD in China was 10.8% in adults and was even higher (14.2%) in Central China^2^. Chronic glomerulonephritis (GN) remains the most common cause of end-stage renal disease (ESRD) in most developing countries, including China^3^. It is also the most common cause of kidney disease in hospitalized patients undergoing renal biopsy. In China, the percentage of hospitalized patients with CKD has increased from 3.58% in 2010 to 4.95% in 2017^3^.

The pathogenesis of CKD is complex and involves numerous factors including genetics, epigenetics, environmental exposures, and behavioral/lifestyle factors. For instance, IgA nephropathy (IgAN) and focal segmental glomerulosclerosis (FSGS) remain the most common GNs in European adult populations, while IgAN and lupus nephritis (LN) are the most common in Asia^4^, LN and FSGS predominate in Latin America^4^, and membranoproliferative glomerulonephritis (MPGN) is most common in South Africa^5^. FSGS was the most common pathological finding in the United States^6^, but its frequency has declined during the past decade, and the frequency of diabetic kidney disease has increased dramatically over the past 30 years. In East China, IgAN continued to be the most common primary glomerulonephritis (PGN) based on 40,759 biopsy-proven cases between 2007 and 2016^7^, while membranous nephropathy (MN) has been the most common pathological finding in a study of 2,725 cases in Northeast China^8^. It is therefore essential to understand the epidemiological trend of renal disease in different ethnic groups and geographic regions. This information will shine new light on the pathogenesis of various renal diseases and provide a basis for further hypothesis-driven research. Central China has among the highest prevalence of renal disease in China. However, contemporary large-scale epidemiological studies of renal disease from this region are lacking. Accordingly, we retrospectively analyzed 34,630 hospitalized patients who underwent native renal biopsy in our center during the past 10 years to explore the epidemiological trend and the prevalence of biopsy-proven renal diseases in this region.

## Study design and methods

In this cross-sectional retrospective study, we enrolled 34,839 patients who underwent native renal biopsy from our center, which covers more than 15 cities in Henan Province in Central China, during the period of January 1, 2009 to December 31, 2018. 209 cases with incomplete general data or insufficient renal tissue for evaluation were excluded; therefore, 34,630 eligible cases were included. Patients who had multiple biopsies, only the first biopsy was used in this study. All biopsy specimens were routinely processed and evaluated by light and immunofluorescence microscopy. Since 2014, electron microscopy (EM) was performed on nearly all biopsies. Before 2014, due to lack of EM facility in our center, EM was performed at an outside facility mainly on cases without which a definitive diagnosis cannot be reached (such as MCD, suspected hereditary and congenital renal disease and so on). Overall, 60.69% of the cohort were examined by EM. Congo red staining was performed in patients over 40 years old (48.34%). All specimens were stained with hematoxylin and eosin (H&E), periodic acid-Schiff (PAS), Masson trichrome, and methenamine silver-periodic acid for light microscopy. Immunofluorescence staining included IgG, IgM, IgA, C3, C4, C1q, fibrinogen, and both kappa and lambda light chains (except children ≤ 14 years old). In cases with positive IgG, IgG subclass staining was performed as well.

The clinical indications for biopsy were classified as follows: nephrotic syndrome (NS), nephritic syndrome, acute nephritic syndrome (ANS), rapidly progressive glomerulonephritis (RPGN), asymptomatic urinary abnormalities (AUA), acute kidney injury (AKI) and chronic kidney disease (CKD). The definition of various clinical syndromes was listed in Table 1.

**Table 1:** The definition of various clinical indications.

Clinical indications	Definition
NS	24-h urine protein ≥ 3.5 g with edema, accompanied by serum albumin < 30 g/L, hyperlipidemia
Nephritic syndrome	24-h urine protein between 1.5 and 3.5 g with hematuria, with or without high blood pressure and edema
ANS	Abrupt onset of hematuria, hypertension, edema, oliguria and reduced eGFR
RPGN	Acute nephritic syndrome with acute deteriorated renal function such as a twofold increase in serum creatinine concentration or a decrease in creatinine clearance by 50%
AUA	Microscopic hematuria and /or subnephrotic proteinuria with no clinical symptoms or signs
AKI	SCr increase ≥ 0.3 mg/dl (≥ 26.5 umol/L) within 48 h or SCr increased by ≥ 1.5 times the baseline value within the prior 7 days or urinary output < 0.5 ml/kg/h for 6 h
CKD	eGFR < 60 mL/min/1.73 m^2^ or > 60 mL/min/1.73 m^2^ with urine albumin creatinine ratio (UACR) > 3 mg/mmol for ≥ 3 months

*NS* nephrotic syndrome, *ANS* acute nephritic syndrome, *RPGN* rapidly progressive renal glomerulonephritis, *AUA* asymptomatic urinary abnormalities, *AKI* acute kidney injury, *CKD* chronic kidney disease.

Histopathological findings were organized according to recent classification system^9^. However, the terms of primary and secondary GN were kept in order to facilitate comparison with historical literatures. Briefly, the morphologic data were organized as follows: (1) primary GN (PGN), including MN, IgAN, minimal change disease (MCD), FSGS, MPGN (referring to Ig-mediated MPGN excluding identifiable etiologies such as chronic infections, autoimmune diseases, dysproteinemias, among others), C3 glomerulopathy, and postinfectious GN, as well as MN combined with IgAN and MCD combined with IgAN, the latter two of which were detailed in previous reports^10,11^; (2) systemic/secondary GN (SGN), including LN, Henöch–Schönlein purpura nephritis (HSPN), hepatitis B associated glomerulonephritis (HBV-GN, defined as HBV-DNA replication without SLE, and with HBsAg and/or HBcAg deposition in glomerulus), diabetic nephropathy (DN), obesity-related glomerulopathy (ORG), paraprotein-associated glomerulopathy {paraprotein-GN, including amyloidosis, monoclonal immunoglobulin deposition diseases (MIDD) and proliferative GN with monoclonal immunoglobulin deposition (PGNMID)}, pauci-immune/ANCA-associated GN (pauci-immune GN), and anti-GBM nephritis (anti-GBM); (3) tubulointerstitial diseases (TID), including acute interstitial nephritis (AIN), acute tubular necrosis (ATN), chronic tubulointerstitial nephritis (CTIN), and light chain cast nephropathy; (4) vascular diseases, including hypertensive nephrosclerosis and thrombotic microangiopathy (TMA); (5) hereditary and congenital renal disease, such as thin basement membrane nephropathy, Alport syndrome, Fabry disease, and lipoprotein glomerulopathy, among others; and (6) miscellaneous, including entities that were difficult to classify according to the current classification scheme and rare entities.

Clinical and pathological diagnoses of all cases had been established by a consensus among experienced nephrologists ​and nephropathologists. The study protocol was approved by the Ethics Committee of The First Affiliated Hospital of Zhengzhou University. All methods were carried out in accordance with relevant guidelines and regulations. Informed consent was obtained from all subjects or, if subjects are under 18, from a parent and/or legal guardian.

### Statistical analysis

SPSS software was used for statistical analysis and the values were expressed as mean ± SD. Paired means were analyzed using Student’s two-tailed t-test. Statistically significant difference was set at *P* < 0.05, and highly significant difference was set at *P* < 0.01.

## Results

### Clinical information

**Figure 1:**
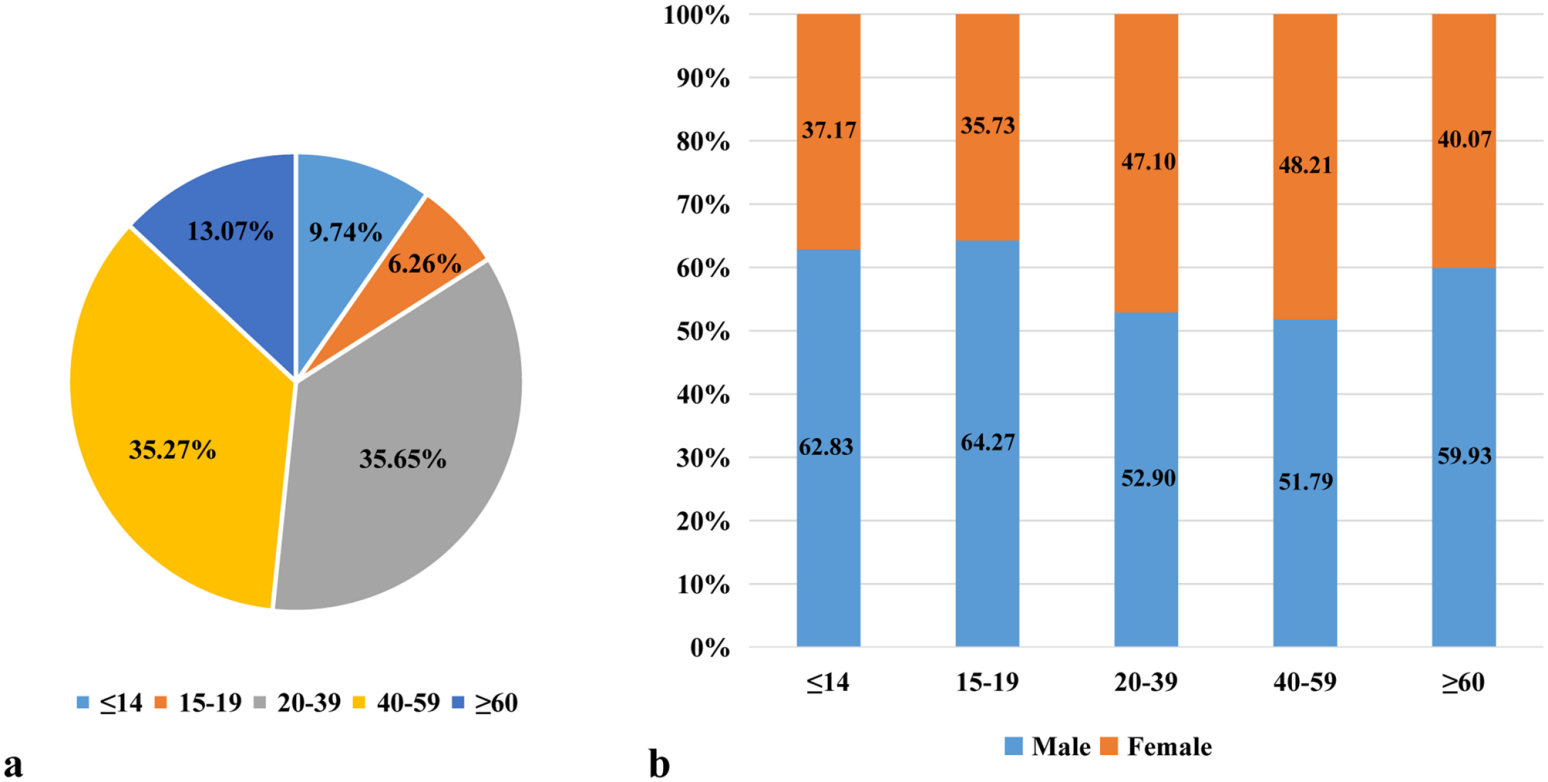
The distribution of renal biopsy based on different age groups and genders. (**a**) The distribution of renal biopsy based on different age groups. (**b**) The gender distributions of renal biopsy based on age.

A total of 34,630 cases between January 1, 2009 and December 31, 2018 were analyzed. The age at the time of renal biopsy varied from 8 months to 90 years, with a male (19,084 cases, 55.11%) to female (15,546 cases, 44.89%) ratio of 1.23:1. Pediatric patients (≤ 14 years old) accounted for 9.74% (3,374 cases) of the full cohort, with mean age at the time of biopsy 8.81 ± 3.34 years, and a male (62.83%) to female (37.17%) ratio of 1.69:1. Adult patients accounted for 90.26% (31,256 cases) of all cases, with mean age at the time of biopsy 40.86 ± 15.28 years, and a male (54.27%) to female (45.73%) ratio of 1.19:1. Among the adult subjects, elderly patients (≥ 60 years) accounted for 13.07% (4,527 cases) of the full cohort , with a mean age at the time of biopsy 66.16 ± 5.25 years, and a ratio of male (59.93%) to female (40.07%) patients of 1.50:1 (Fig. 1a, b).

**Figure 2:**
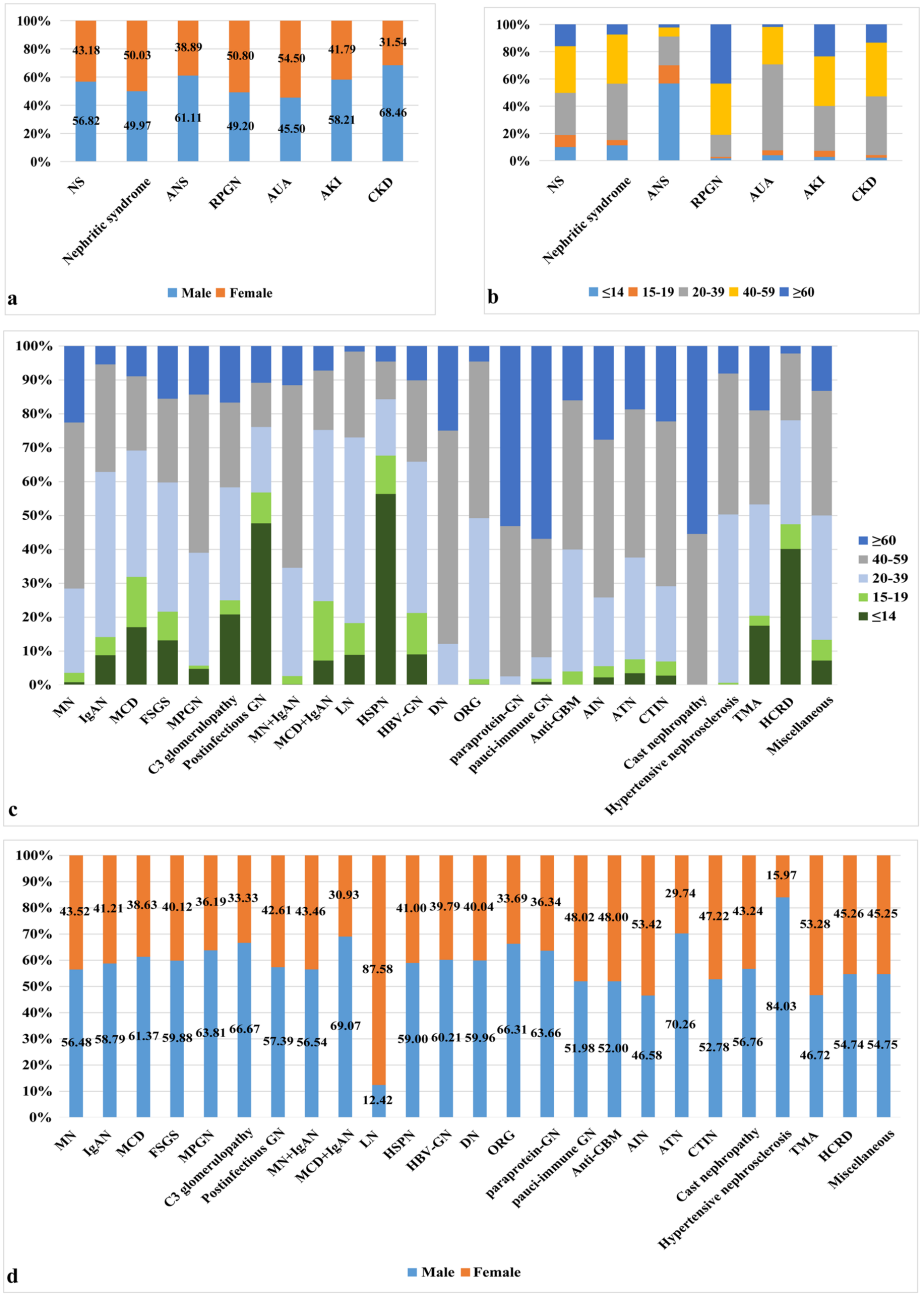
The gender and age distributions according to clinical indications for biopsy and pathological findings. (**a**) The gender distributions according to clinical indications. (**b**) The age distributions according to clinical indications. (**c**) The age distributions according to pathological findings. (**d**) The gender distributions according to pathological findings (*cast nephropathy* light chain cast nephropathy, *HCRD* hereditary and congenital renal disease).

Nephrotic syndrome was the most common clinical indication for renal biopsy, accounting for 51.80% of all cases, followed by nephritic syndrome (36.12%), CKD (7.80%), AKI (1.93%), RPGN (1.44%), AUA (0.64%), and ANS (0.26%). NS was the most common indication for biopsy across all age groups and in both genders, followed by nephritic syndrome. All clinical indications were noted in patients over 20 years of age except ANS, which mainly occurred in children (56.67%). The majority of AUA was seen in young patients, with 63.06% in the 20–39-year age group (Table 2 and Fig. 2a, b).

**Table 2:** Age stratification of patients with biopsy proven renal disease according to their clinical indications.

Clinical indications	Age (years old)					
	≤ 14, N (%)	15–19, N (%)	20–39, N (%)	40–59, N (%)	≥ 60, N (%)	Total, N (%)
NS	1,806 (53.53)	1,574 (72.57)	5,558 (45.02)	6,137 (50.24)	2,863 (63.24)	17,938 (51.80)
Nephritic syndrome	1,420 (42.09)	486 (22.41)	5,166 (41.85)	4,513 (36.95)	924 (20.41)	12,509 (36.12)
ANS	51 (1.51)	12 (0.55)	19 (0.15)	6 (0.05)	2 (0.04)	90 (0.26)
RPGN	9 (0.27)	5 (0.23)	81 (0.66)	188 (1.54)	217 (4.79)	500 (1.44)
AUA	9 (0.27)	8 (0.37)	140 (1.13)	61 (0.50)	4 (0.09)	222 (0.64)
AKI	19 (0.56)	30 (1.38)	220 (1.78)	244 (2.00)	157 (3.47)	670 (1.93)
CKD	60 (1.78)	54 (2.49)	1,161 (9.40)	1,066 (8.73)	360 (7.95)	2,701 (7.80)
Total,N (%)	3,374 (100)	2,169 (100)	12,345 (100)	12,215 (100)	4,527 (100)	34,630 (100)

### Pathological diagnosis

PGN was the most common pathological finding, accounting for 65.06% (22,529 cases), followed by SGN (22.01%, 7,622 cases), TID (2.55%, 884 cases), vascular diseases (1.72%, 594 cases), and hereditary and congenital renal disease (0.40%, 137 cases) (Table 3).

**Table 3:** The distribution of pathological findings based on gender and different age groups.

Histologic diagnosis	Age (years old)					
	≤ 14, N (%)	15–19, N (%)	20–39, N (%)	40–59, N (%)	≥ 60, N (%)	Total, N (%)
**PGN (22,529, 65.06%)**						
MN	72 (2.13)	240 (11.07)	2,149 (17.41)	4,235 (34.67)	1,948 (43.03)	8,644 (24.96)
IgAN	735 (21.78)	447 (20.61)	4,064 (32.92)	2,647 (21.67)	450 (9.94)	8,343 (24.09)
MCD	633 (18.76)	551 (25.40)	1,383 (11.20)	813 (6.66)	330 (7.29)	3,710 (10.71)
FSGS	112 (3.32)	72 (3.32)	324 (2.62)	210 (1.72)	132 (2.92)	850 (2.45)
MPGN	10 (0.30)	2 (0.09)	70 (0.57)	98 (0.80)	30 (0.66)	210 (0.61)
C3 glomerulopathy	10 (0.30)	2 (0.09)	16 (0.13)	12 (0.10)	8 (0.18)	48 (0.14)
Postinfectious GN	84 (2.49)	16 (0.74)	34 (0.28)	23 (0.19)	19 (0.42)	176 (0.51)
MN + IgAN	1 (0.03)	11 (0.51)	144 (1.17)	243 (1.99)	52 (1.15)	451 (1.30)
MCD + IgAN	7 (0.21)	17 (0.78)	49 (0.40)	17 (0.14)	7 (0.15)	97 (0.28)
**SGN (7,622, 22.01%)**						
LN	233 (6.91)	245 (11.30)	1,433 (11.61)	663 (5.43)	42 (0.93)	2,616 (7.55)
HSPN	1,078 (31.95)	216 (9.96)	318 (2.58)	213 (1.74)	87 (1.92)	1,912 (5.52)
HBV-GN	86 (2.55)	116 (5.35)	424 (3.43)	228 (1.87)	96 (2.12)	950 (2.74)
DN	0 (0)	1 (0.05)	113 (0.92)	591 (4.84)	234 (5.17)	939 (2.71)
ORG	1 (0.03)	7 (0.32)	220 (1.78)	214 (1.75)	21 (0.46)	463 (1.34)
paraprotein-GN	0 (0)	0 (0)	10 (0.08)	172 (1.41)	206 (4.55)	388 (1.12)
pauci-immune GN	3 (0.09)	3 (0.14)	21 (0.17)	115 (0.94)	187 (4.13)	329 (0.95)
Anti-GBM	0 (0)	1 (0.05)	9 (0.07)	11 (0.09)	4 (0.09)	25 (0.07)
**TID (884, 2.55%)**						
AIN	9 (0.27)	13 (0.60)	80 (0.65)	184 (1.51)	109 (2.41)	395 (1.14)
ATN	12 (0.36)	14 (0.65)	103 (0.83)	150 (1.23)	64 (1.41)	343 (0.99)
CTIN	2 (0.06)	3 (0.14)	16 (0.13)	35 (0.29)	16 (0.35)	72 (0.21)
Cast nephropathy	0 (0)	0 (0)	0 (0)	33 (0.27)	41 (0.91)	74 (0.21)
**Vascular diseases (594, 1.72%)**						
Hypertensive nephrosclerosis	0 (0)	3 (0.14)	227 (1.84)	190 (1.56)	37 (0.82)	457 (1.32)
TMA	24 (0.71)	4 (0.18)	45 (0.36)	38 (0.31)	26 (0.57)	137 (0.40)
HCRD	55 (1.63)	10 (0.46)	42 (0.34)	27 (0.22)	3 (0.07)	137 (0.40)
Miscellaneous	207 (6.14)	175 (8.07)	1,051 (8.51)	1,053 (8.62)	378 (8.35)	2,864 (8.27)
Total	3,374 (100)	2,169 (100)	12,345 (100)	12,215 (100)	4,527 (100)	34,630 (100)

*PGN* primary glomerulonephritis, *SGN* secondary glomerulonephritis, *TID* tubulointerstitial diseases, *HCRD* hereditary and congenital renal disease, *MN* membranous nephropathy, *IgAN* IgA nephropathy, *MCD* minimal change disease, *FSGS* focal segmental glomerulosclerosis, *MPGN* membranoproliferative glomerulonephritis, *MN* + *IgAN* MN combined with IgAN, *MCD* + *IgAN* MCD combined with IgAN, *LN* lupus nephritis, *HSPN* Henöch–Schönlein purpura nephritis, *HBV-GN* hepatitis B virus associated glomerulonephritis, *DN* diabetic nephropathy, *ORG* obesity-related glomerulopathy, *paraprotein-GN* paraprotein-associated glomerulopathy, *pauci-immune GN* pauci-immune/ANCA-associated glomerulonephritis, *Anti-GBM* anti-GBM nephritis, *AIN* acute interstitial nephritis, *ATN* acute tubular necrosis, *CTIN* chronic tubulointerstitial nephritis. Cast nephropathy, light chain cast nephropathy, *TMA* thrombotic microangiopathy.

MN (24.96%) and IgAN (24.09%) were the leading diagnoses in PGN, followed by MCD (10.71%), FSGS (2.45%), MPGN (0.61%), postinfectious GN (0.51%) and C3 glomerulopathy (0.14%) including C3GN (44 cases) and dense deposit disease (DDD, 4 cases) (Table 3).

In adults, MN and MPGN had distribution peaks in the 40–59-year age group (48.99% and 46.67%, respectively); IgAN, MCD, FSGS and C3 glomerulopathy had distribution peaks in the 20–39-year age group (48.71%, 37.28%, 38.12%, and 33.33%, respectively), and postinfectious GN had distribution peak in children (≤ 14-year age group, 47.73%) (Fig. 2c). In elderly patients, MN (43.03%) was the most common PGN, followed by IgAN (9.94%), MCD (7.29%), FSGS (2.92%), MPGN (0.66%), postinfectious GN (0.42%) and C3 glomerulopathy (0.18%). In children, IgAN (21.78%) was the most common PGN, followed by MCD (18.76%), FSGS (3.32%), postinfectious GN (2.49%), MN (2.13%), MPGN (0.30%) and C3 glomerulopathy (0.30%) (Table 3). In our center, 451 cases of combined MN with IgAN (1.30%) were diagnosed, mainly in the 40–59-year age group (53.88%), similar to the distribution seen in MN. MCD combined with IgAN, accounting for 0.28% (97 cases), peaked in the 20–39-year age group (50.52%) (Table 3 and Fig. 2c). Since 2014, PLA2R staining has been done for 2,593 cases of MN, 84.65% (2,195 cases) of which were positive. In addition, IgG subclass staining was also performed in these patients. IgG4 was the predominate staining in 90.12% of cases, followed by IgG1 (9.25%), IgG3 (0.50%) and IgG2 (0.13%).

LN (7.55%) was the most common SGN, followed by HSPN (5.52%), HBV-GN (2.74%), DN (2.71%), ORG (1.34%), paraprotein-GN (1.12%, including 361 cases of amyloidosis, 17 cases of MIDD, and 10 cases of PGNMID), pauci-immune GN (0.95%), and anti-GBM (0.07%) (Table 3). Based on the ISN/RPS classification, the cohort of LN can be divided as follows: class I—0.96% ( 25 cases); class II—6.77% (177 cases); class III—15.52% (406 cases); class III & V—11.96% (313 cases); class IV—36.89% (965 cases); class IV & V—15.44% (404 cases); class V—12.35% (323 cases); and class VI—0.11% (3 cases). In adults, LN had a distribution peak in the 20–39-year age group (54.78%) and was more prevalent in females (87.58%). DN peaked in the 40–59-year age group (62.94%), while pauci-immune GN was more commonly seen in elderly patients (56.84%), and HBV-GN was most prevalent in the 20–39-year age group (44.63%). In elderly patients, DN (5.17%), paraprotein-GN (4.55%), and pauci-immune GN (4.13%) were the leading diagnoses in SGN. In children, HSPN (31.95%) was the most common SGN, followed by LN (6.91%) (Table 3 and Fig. 2c, d).

For tubulointerstitial diseases, AIN accounted for 1.14%, followed by ATN (0.99%), CTIN (0.21%), and light chain cast nephropathy (0.21%). Regarding vascular diseases, hypertensive nephrosclerosis accounted for 1.32% and TMA for 0.40%. Hereditary and congenital renal disease was diagnosed in 137 cases (0.40%), and mainly occurred in children (40.15%) (Table 3 and Fig. 2c).

### Clinicopathologic correlations

MN was the most common glomerulopathy in patients with NS (38.18%), followed by MCD (20.15%) and IgAN (11.13%). Among the patients presenting with nephritic syndrome, IgAN was the most common pathological finding (39.69%), followed by MN (13.69%) and LN (11.10%). Postinfectious GN was the most common pathological finding in patients presenting with ANS (46.67%), followed by IgAN (38.89%). Pauci-immune GN was the most common cause of RPGN (50.20%), followed by AIN (11.60%). Among patients presenting with AKI, ATN and AIN were the leading diagnoses (33.88% and 33.13%, respectively). IgAN was the most common pathological finding in patients presenting with AUA and CKD (50.45% and 42.21%, respectively) (Table 4).

**Table 4:** The clinicopathological findings of renal biopsy based on different clinical indications.

Pathological diagnosis	Clinical indications							
	NS, N (%)	Nephritic syndrome, N (%)	ANS, N (%)	RPGN, N (%)	AUA, N (%)	AKI, N (%)	CKD, N (%)	Total, N (%)
MN	6,848 (38.18)	1,712 (13.69)	7 (7.78)	6 (1.20)	22 (9.91)	4 (0.60)	45 (1.67)	8,644 (24.96)
IgAN	1,996 (11.13)	4,965 (39.69)	35 (38.89)	35 (7.00)	112 (50.45)	60 (8.96)	1,140 (42.21)	8,343 (24.09)
MCD	3,615 (20.15)	80 (0.64)	0 (0)	1 (0.20)	0 (0)	7 (1.04)	7 (0.26)	3,710 (10.71)
FSGS	613 (3.42)	164 (1.31)	1 (1.11)	0 (0)	2 (0.90)	7 (1.04)	63 (2.33)	850 (2.45)
MPGN	109 (0.61)	91 (0.73)	0 (0)	0 (0)	0 (0)	0 (0)	10 (0.37)	210 (0.61)
C3 glomerulopathy	17 (0.09)	17 (0.14)	1 (1.11)	3 (0.60)	2 (0.90)	2 (0.30)	6 (0.22)	48 (0.14)
Postinfectious GN	59 (0.33)	59 (0.47)	42 (46.67)	3 (0.60)	2 (0.90)	5 (0.75)	6 (0.22)	176 (0.51)
MN + IgAN	348 (1.94)	101 (0.81)	0 (0)	0 (0)	0 (0)	0 (0)	2 (0.07)	451 (1.30)
MCD + IgAN	92 (0.51)	5 (0.04)	0 (0)	0 (0)	0 (0)	0 (0)	0 (0)	97 (0.28)
LN	1,120 (6.24)	1,389 (11.10)	0 (0)	18 (3.60)	0 (0)	18 (2.69)	71 (2.63)	2,616 (7.55)
HSPN	753 (4.20)	956 (7.64)	0 (0)	0 (0)	29 (13.06)	0 (0)	174 (6.44)	1,912 (5.52)
HBV-GN	794 (4.43)	146 (1.17)	1 (1.11)	0 (0)	3 (1.35)	0 (0)	6 (0.22)	950 (2.74)
DN	562 (3.13)	199 (1.59)	0 (0)	0 (0)	15 (6.76)	6 (0.90)	157 (5.81)	939 (2.71)
ORG	16 (0.09)	424 (3.39)	0 (0)	0 (0)	4 (1.80)	0 (0)	19 (0.70)	463 (1.34)
paraprotein-GN	295 (1.64)	71 (0.57)	0 (0)	0 (0)	1 (0.45)	1 (0.15)	20 (0.74)	388 (1.12)
pauci-immune GN	12 (0.07)	27 (0.22)	2 (2.22)	251 (50.20)	0 (0)	8 (1.19)	29 (1.07)	329 (0.95)
Anti-GBM	1 (0.006)	3 (0.02)	0 (0)	13 (2.60)	0 (0)	1 (0.15)	7 (0.26)	25 (0.07)
AIN	1 (0.006)	22 (0.18)	0 (0)	58 (11.60)	0 (0)	222 (33.13)	92 (3.41)	395 (1.14)
ATN	0 (0)	40 (0.32)	0 (0)	14 (2.80)	0 (0)	227 (33.88)	62 (2.30)	343 (0.99)
CTIN	0 (0)	6 (0.05)	0 (0)	0 (0)	0 (0)	4 (0.60)	62 (2.30)	72 (0.21)
Cast nephropathy	4 (0.02)	15 (0.12)	0 (0)	2 (0.40)	0 (0)	10 (1.49)	43 (1.59)	74 (0.21)
Hypertensive nephrosclerosis	18 (0.10)	211 (1.69)	0 (0)	4 (0.80)	0 (0)	20 (2.99)	204 (7.55)	457 (1.32)
TMA	26 (0.14)	59 (0.47)	1 (1.11)	0 (0)	0 (0)	16 (2.39)	35 (1.30)	137 (0.40)
HCRD	23 (0.13)	97 (0.78)	0 (0)	0 (0)	0 (0)	0 (0)	17 (0.63)	137 (0.40)
Miscellaneous	616 (3.43)	1,650 (13.19)	0 (0.00)	92 (18.40)	30 (13.51)	52 (7.76)	424 (15.70)	2,864 (8.27)
Total (%)	17,938 (100)	12,509 (100)	90 (100)	500 (100)	222 (100)	670 (100)	2,701 (100)	34,630 (100)

**Figure 3:**
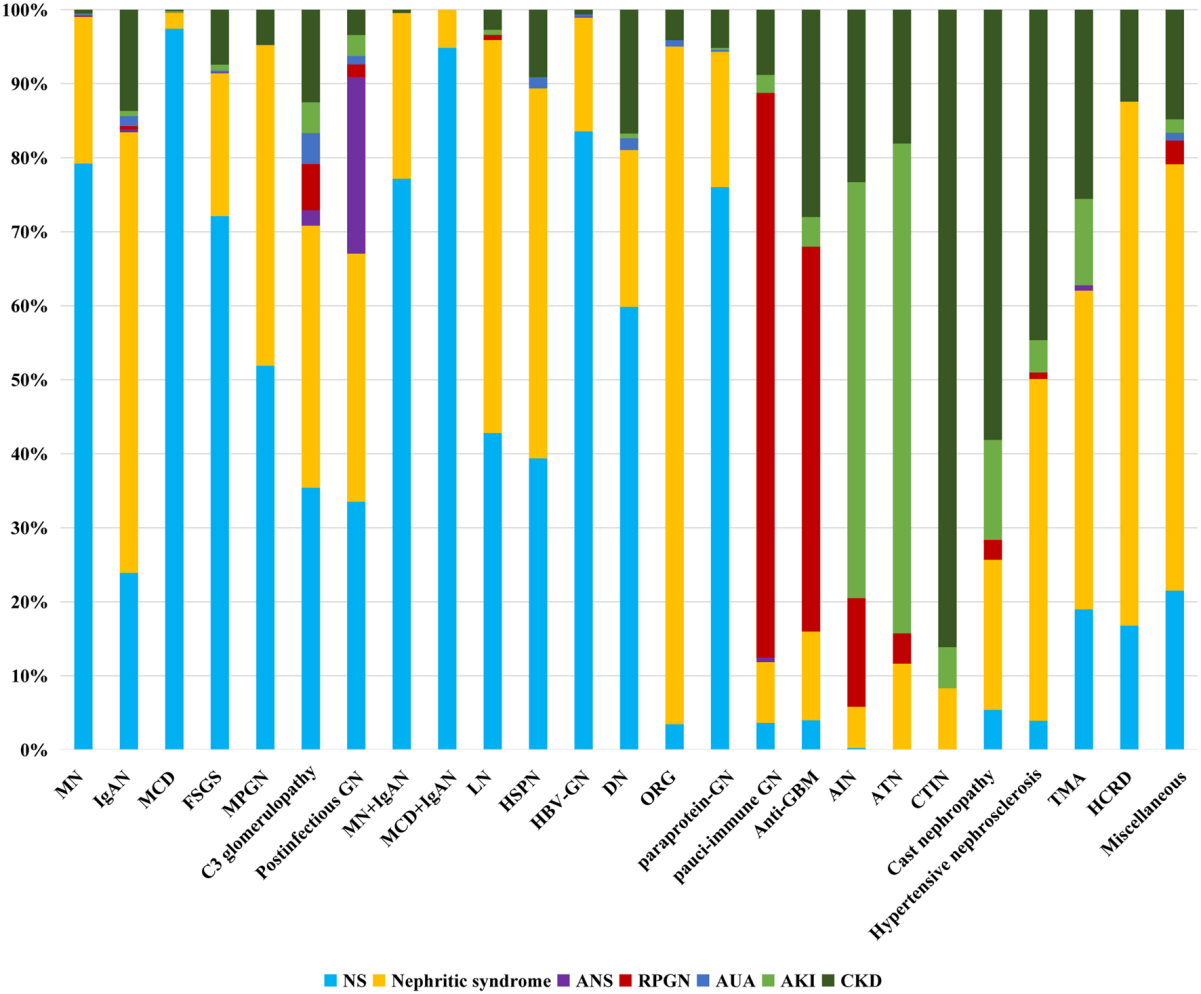
Clinicopathological correlations (*Cast nephropathy* light chain cast nephropathy, *HCRD* hereditary and congenital renal disease).

Regarding primary GN, NS was the most common clinical manifestation in patients with MN (79.22%), followed by nephritic syndrome (19.81%), while nephritic syndrome was most common in IgAN (59.51%), followed by NS (23.92%). In MCD, 97.44% patients presented with NS. In FSGS, NS was the most common clinical presentation (72.12%), followed by nephritic syndrome (19.29%). In MPGN, NS and nephritic syndrome were the leading clinical manifestations, accounting for 51.90% and 43.33%, respectively. Similarly, C3 glomerulopathy mostly manifested as NS (35.42%) and nephritic syndrome (35.42%). In postinfectious GN, NS (33.52%) and nephritic syndrome (33.52%) were the most common clinical presentation, followed by ANS (23.86%). NS was the most common clinical presentation in both MN combined with IgAN (77.16%) and MCD combined with IgAN (94.85%). In secondary GN, nephritic syndrome was the leading clinical indication in LN (53.10%), HSPN (50.00%), and ORG (91.58%), while NS was more common in DN (59.85%), paraprotein-GN (76.03%), and HBV-GN (83.58%). RPGN was the most common manifestation of both pauci-immune GN (76.29%) and anti-GBM (52.00%). In ATN and AIN, AKI was the most common clinical presentation, accounting for 66.18% and 56.20%, respectively. CTIN and light chain cast nephropathy presented clinically with CKD in 86.11% and 58.11% of cases, respectively. Nephritic syndrome was the leading clinical indication in vascular diseases, as well as in hereditary and congenital renal disease (70.80%) (Fig. 3).

### Temporal and demographic trends in renal disease frequencies

**Figure 4:**
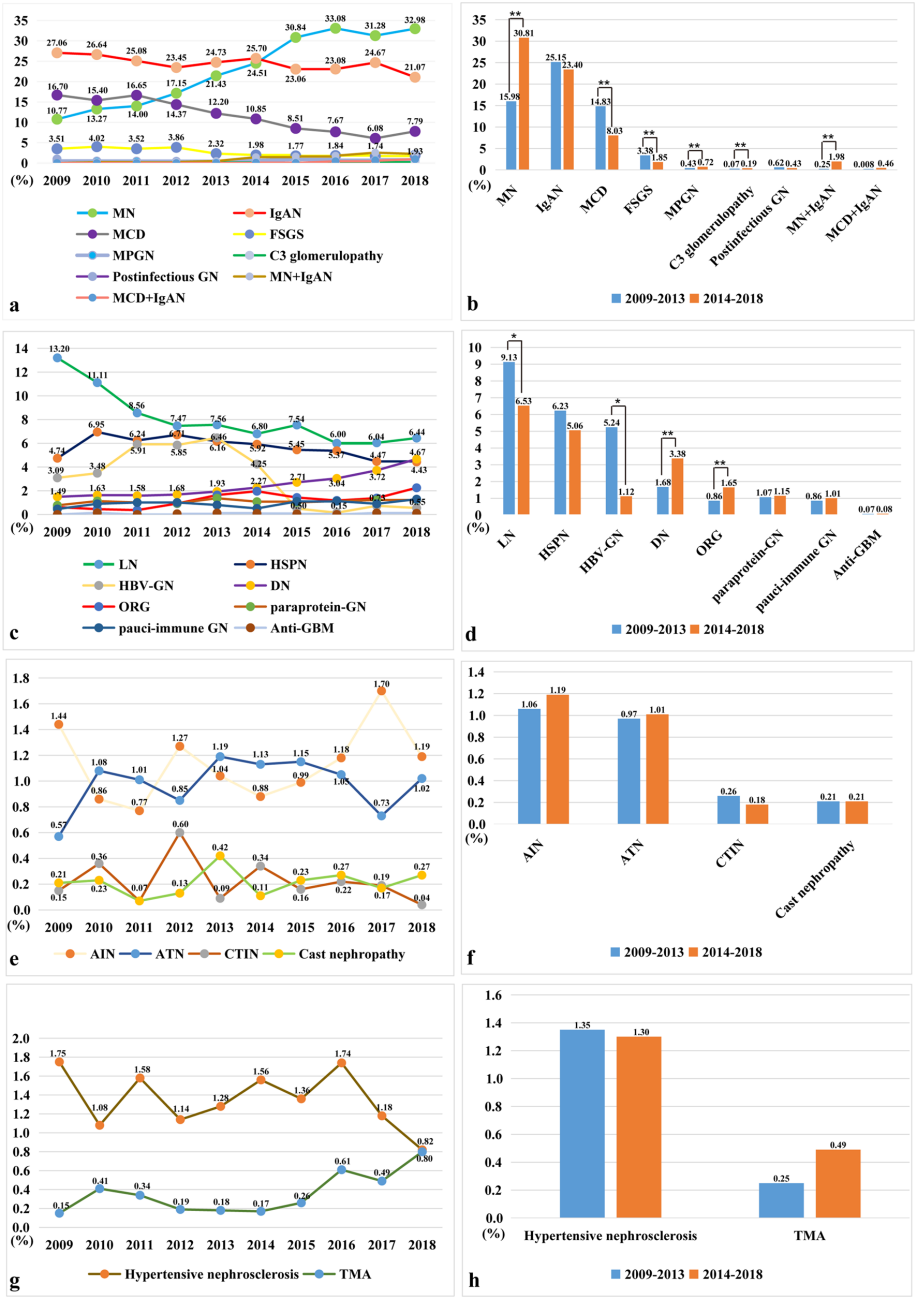
Annual prevalence of various pathological findings and the temporal trends in disease frequencies. (**a**) The annual prevalence of PGN. (**b**) The changing prevalence of PGN according to periods. (**c**) The annual prevalence of SGN. (**d**) The changing prevalence of SGN according to periods. (**e**) The annual prevalence of TID. (**f**) The changing prevalence of TID according to periods. (**g**) The annual prevalence of vascular diseases. (**h**) The changing prevalence of vascular diseases according to periods.*P < 0.05, **P < 0.01 (*Cast nephropathy* light chain cast nephropathy, *HCRD* hereditary and congenital renal disease).

To analyze the temporal and demographic trends, we further divided the study duration into 2 periods, 2009 to 2013 (period 1) and 2014 to 2018 (period 2). The frequency of MN in renal biopsy specimens increased strikingly, from 10.77% in 2009 to 32.98% in 2018, and has overtaken IgAN as the leading diagnosis since 2015 (Fig. 4a). The prevalence of MN nearly doubled from 2009–2013 (15.98%) to 2014–2018 (30.81%) (*P* = 0.0004) (Fig. 4b). The same trend appeared with MPGN (0.43% vs 0.72%, *P* = 0.0042) and C3 glomerulopathy (0.07% vs 0.19%, *P* = 0.0081) , while the frequencies of MCD (14.83% vs 8.03%, *P* = 0.0011) and FSGS (3.38% vs 1.85%, *P* = 0.0084) decreased significantly between the two intervals (Fig. 4b). No significant changes were noted for IgAN and postinfectious GN. In our center, MN combined with IgAN was first diagnosed in 2010, the frequency of which increased significantly over time (*P* = 0.0005), from 0.25% in period 1 to 1.98% in period 2. MCD combined with IgAN was first diagnosed in 2013 and increased from 0.03% in 2013 to 0.96% in 2018 (Fig. 4a, b).

Regarding secondary GN, the frequency of LN decreased significantly over time, from 13.20% in 2009 to 6.44% in 2018, while DN (1.49% vs 4.67%) and ORG (0.62% vs 2.25%) more than tripled during the two time intervals (Fig. 4c). Between period 1 and period 2, the frequency of LN (9.13% vs 6.53%, *P* = 0.0338), and HBV-GN (5.24% vs 1.12%, *P* = 0.0487) decreased significantly, while DN (1.68% vs 3.38%, *P* = 0.0098) and ORG (0.86% vs 1.65%, *P* = 0.0054) increased with statistical significance between the two intervals. No significant changes were noted for HSPN, paraprotein-GN, pauci-immune GN, and anti-GBM (Fig. 4d). As shown in Fig. 4e–h, there was no significant change in the frequencies of TID and vascular diseases between the two intervals. The category of miscellaneous diseases decreased significantly (9.98% vs 7.64%, *P* = 0.0319).

## Discussion

Currently, there are no contemporary large-scale renal biopsy studies addressing kidney disease epidemiology in Central China. Henan province, located in Central China, is one of the most populated provinces in China, with over 100 million people, and has among the highest prevalence of renal disease in China. As one of the largest kidney pathology centers in China (located in Zhengzhou, the capital of Henan province), we retrospectively analyzed 34,630 hospitalized patients residing in Henan province who underwent native renal biopsy in our center over the past 10 years to explore the epidemiology of kidney disease. This study provides for the first time a picture of the current spectrum of renal disease in this region, which can serve as a useful resource for making health policy decisions and hypothesis-driven research to conquer renal diseases.

We identified primary GN as the leading pathological findings, followed by systemic/secondary GN (Table 3). A significant temporal shift in the epidemiology of many renal diseases over the past decade was noted. In adults, the prevalence of MN increased dramatically over the past decade. The frequency of MN increased from 10.77% in 2009 to 32.98% in 2018, and MN has overtaken IgAN as the most common PGN since 2015 (Fig. 4a). The prevalence nearly doubled from 2009–2013 (15.98%) to 2014–2018 (30.81%) (*P* = 0.0004) (Fig. 4b), which is consistent with previous studies from Northeast China^8^. The prevalence of MN has also increased significantly in India^12^ and Brazil^13^, but has decreased in Japan^14^, Korea^15^, USA^16,17^, and UK^18^. It is suggested that the increased prevalence of MN in developing countries, including China, and its decrease in developed countries might be related to environmental exposures related to industrialization, as well as socioeconomic factors and geographical and genetic influences^7,19^. In fact, Xu et al. found long-term exposure to air pollution to be associated with increased risk of MN in China^20^. Since 2014, PLA2R staining has been done for 2,593 cases of MN, 84.65% (2,195 cases) of which were positive, which is similar to the findings previously reported (85%)^21^.

In our study, IgAN showed a gradually decreasing trend for the study duration, with 27.06% in 2009 and 21.07% in 2018, but there is no statistical significance between the two intervals (*P* = 0.1036) (Fig. 4a, b), and this phenomenon differs from other Asian countries. For instance, the relative frequency of IgAN has increased significantly in Korea^15^. In period 1, IgAN (25.15%) was the most common PGN, which is consistent with previous studies by Xu et al. from Central China among 4,931 cases from 1994 to 2014^22^, Zhou et al. from East China among 10,779 cases from 2001 to 2015^23^, and Hou et al., also from East China, among 40,759 cases from 2003 to 2014^7^ in the same interval.

The clinical indications for renal biopsy in IgAN had some changes over the past decade (Supplementary Fig. S online). AUA decreased significantly (*P* = 0.0256) while CKD increased significantly (*P* = 0.0331) between the two intervals. Nephritic syndrome remained the most common clinical indication for biopsy in IgAN and had no significant changes (*P* = 0.6250) between the two intervals. NS, ANS, RPGN, and AKI had a mild decrease without statistical significance (*P* = 0.1137, 0.2161, 0.8093, and 0.6761, respectively) between the two intervals.

MCD contributed 10.71% in our study, within the range seen in other areas of China (8.1–14.81%)^7,8,19,23,24^, but significantly higher than those previously reported in Central China (1.82%) by Xu et al.^22^. The frequency gradually decreased during the study period, with statistical significance between the two intervals (14.83% vs 8.03%, *P* = 0.0011) (Fig. 4a, b). These findings are consistent with previous studies by Zhu et al. from Beijing^19^, and Hou et al. from East China^7^ in the same interval, as well as other Asian countries including Korea^15^, but are opposite to findings reported by Xu et al. from Central China^22^, Su et al. from Northeast China^8^, and Zhou et al. from East China^23^.

The frequency of FSGS was 2.45%, similar to that reported in other centers of China^8,22,23^, but significantly lower than that reported (7.34%) by Hou et al. from East China^7^. A gradually decreasing trend was shown, with statistical significance between the two intervals (3.38% vs 1.85%, *P* = 0.0084) (Fig. 4a, b). The change in prevalence is consistent with previous studies by Xu et al. from Central China in the same interval^22^, as well as Hou et al. from East China^7^ and Su et al. from Northeast China^8^, but opposite to reports from other centers in China^19^.

Immune complex-mediated MPGN without identifiable etiologies contributed 0.61% in our cohort. The frequency increased significantly between the two intervals (0.43% vs 0.72%, *P* = 0.0042) (Fig. 4a, b), which is opposite to the study by Xu et al. from Central China^22^ and other Asian countries, such as Korea^15^.

In this cohort, 48 cases of C3 glomerulopathy (44 cases of C3GN, 4 cases of DDD) has been diagnosed, representing 0.14% of the full cohort. In C3GN, the morphologic patterns included endocapillary proliferative GN (38.64%, 17 cases), mesangial proliferative GN (25.00%, 11 cases), MPGN (18.18%, 8 cases), necrotizing/crescentic GN (11.36%, 5 cases), FSGS (4.55%, 2 cases), and normal by light microscopy (2.27%, 1 case).

In this study, postinfectious GN, all showed elevated anti-streptolysin O titers, represented 0.51% of the full cohort, which is lower than the previous report (7.5%)^25^. The change of frequency has no statistical significance between the two intervals (*P* = 0.2067). In our center, just few IgA dominant-infection-associated glomerulonephritis (15 cases) was diagnosed, and classified to the cohort of miscellaneous.

Recently, an increasing number of reports have emerged in regard to IgAN combined with other renal diseases^10,11,26,27^. In our study, MN combined with IgAN was diagnosed in 451 cases since 2010, with NS as the most common clinical indication (77.16%) for biopsy, followed by nephritic syndrome (22.39%) (Fig. 3). MCD combined with IgAN has been uncommonly reported, mostly in patients presenting with full NS who meet criteria for IgAN with only mild mesangial proliferation, in addition to diffuse foot process effacement without peripheral capillary wall immune complex deposits, reminiscent of MCD^11^. Since 2013, we have diagnosed 97 cases, mostly presenting with NS (94.85%), followed by nephritic syndrome (5.15%) (Fig. 3).

In adults, LN remains the most common SGN, although prevalence has significantly decreased over time, which is compatible with most studies^7,15,22,23,25,28^, but differs from the report from Northeast China which showed HSPN as the most common SGN followed by LN^8^. Additionally, a significant decrease in the prevalence of HBV-GN (MN pattern: 881 cases, MPGN pattern: 69 cases) was also observed from period 1 to period 2 (5.24% vs 1.12%, *P* = 0.0487)(Table 3 and Fig. 4c, d). This change is related to the widespread HBV vaccinations in China since 1992, leading to marked reduction in HBV infections^8,29^.

The prevalence of DN and ORG has gradually increased with statistical significance between the two intervals (1.68% vs 3.38%, *P* = 0.0098, 0.86% vs 1.65%, *P* = 0.0054, respectively) (Fig. 4c, d), similar to findings regarding DN reported by other centers in China^22,23^. A recent study estimated that high body mass index (BMI) was the leading individual-attributable factor for diabetes mellitus in China, responsible for 43.8 million cases, with a population attributable fraction of 46.8% in 2011^3,30^. Indeed, Chinese men and women ranked 60th and 41st, respectively, in terms of severe obesity in 1975, while both were ranked second globally by 2014^3,31^. DN and ORG are both potentially preventable through promoting healthy lifestyle choices and reducing obesity, which are topics that are at the forefront of current public health campaigns^4^.

TID represented 2.55% of the full cohort, with no significant change in prevalence over the past decade (Table 3), which is consistent with previous studies by Zhou et al. from East China^23^, but contrary to the findings reported by Su et al. from Northeast China^8^, in which the frequency of TID significantly increased. There was no significant change in the prevalence of AIN, ATN, CTIN, or light chain cast nephropathy between the two intervals (Fig. 4e, f).

The frequency of vascular diseases was 1.72% in our study, and there was no significant change in frequency for the study duration. The prevalence of both hypertensive nephrosclerosis and TMA had no significant changes between the two intervals (Fig. 4g–h).

Hereditary and congenital renal disease was diagnosed in 137 cases (0.40%) in our center, mainly distributed in children (40.15%) (Table 3 and Fig. 2c). Alport syndrome contributed 49.64% (68 cases), thin basement membrane nephropathy 16.06% (22 cases), Fabry disease 8.76% (12 cases), and lipoprotein glomerulopathy 8.03% (11 cases).

In children, IgAN (21.78%) was the most common PGN, followed by MCD (18.76%) (Table 3). In comparison, MCD was the most common PGN in children reported by Nie et al. (29%, followed by IgAN)^32^ and in Jordan (27%, followed by FSGS)^33^, while FSGS was the most common in Greece (15%, followed by IgAN)^34^. HSPN (31.95%) was the most common SGN, followed by LN (6.91%) (Table 3), which is compatible with the previous study by Nie et al.^32^ and in Jordan^33^, whereas LN was the most common SGN reported from Greece (8.5%, followed by HSPN)^34^. Focusing on patients ≤ 10 years old (2,332 cases, 6.73%), we found that the frequency of MCD (459 cases, 1.33%) was slightly higher than IgAN (437 cases, 1.26%) in PGN, while HSPN (714 cases, 2.06%) was still the most common SGN, followed by LN (97 cases, 0.28%).

In our study, NS was the most common clinical indication for renal biopsy, followed by nephritic syndrome (Table 2). These results are consistent with most previous reports^12,28,35,36^, including those from several centers in China^7,8,19^, but not those from Japan (nephritic syndrome followed by NS)^37^, South Korea (AUA, followed by NS)^25^, and one report with a smaller number of cases from Central China (proteinuria and hematuria, followed by NS)^22^. Variation in renal biopsy policies and in diagnostic criteria of histopathological diagnoses may be the reasons of the above-mentioned discrepancies.

Central China has among the highest prevalence of renal disease in China, and this is the first large-scale epidemiological study of renal disease from this region. However, as a single-center study, the enrolled patients mostly came from Henan province, and may not represent the variety of regions in China and the Chinese population as a whole. Additionally, there may be bias for the enrolled patients who underwent renal biopsy due to variation in renal biopsy policies and in diagnostic criteria of histopathological diagnoses.

In summary, PGN was the most common pathological finding overall, and MN was the most common PGN in adults, while IgAN was the most common in children. LN was the most common SGN in adults, although the prevalence has significantly decreased over time, while HSPN was the most common SGN in children. DN and ORG tripled over the past decade. Together, these findings describe the local epidemiological trends and the prevalence of renal biopsies in Central China and provide a reference point for better understanding and prevention of renal disease.

## Supplementary information

Supplementary figure S1

Supplementary information

## References

[1] Venuthurupalli SK, Hoy WE, Healy HG, Cameron A, Fassett RG (2017). CKD.QLD: establishment of a Chronic Kidney Disease [CKD] Registry in Queensland, Australia. BMC Nephrol..

[2] Zhang L (2012). Prevalence of chronic kidney disease in China: a cross-sectional survey. Lancet.

[3] Yang C (2019). CKD in China: evolving spectrum and public health implications. Am. J. Kidney Dis..

[4] O'Shaughnessy MM (2018). Glomerular disease frequencies by race, sex and region: results from the international kidney biopsy survey. Nephrol. Dial. Transplant..

[5] Okpechi I (2011). Patterns of renal disease in Cape Town South Africa: a 10-year review of a single-centre renal biopsy database. Nephrol. Dial. Transplant..

[6] Haas M, Spargo BH, Coventry S (1995). Increasing incidence of focal-segmental glomerulosclerosis among adult nephropathies: a 20-year renal biopsy study. Am. J. Kidney Dis..

[7] Hou JH (2018). Changes in the spectrum of kidney diseases: an analysis of 40,759 biopsy-proven cases from 2003 to 2014 in China. Kidney Dis..

[8] Su S (2019). clinicopathologic correlations of renal biopsy findings from Northeast China: a 10-year retrospective study. Medicine.

[9] Haas M, Rastaldi MP, Fervenza FC (2014). Histologic classification of glomerular diseases: clinicopathologic correlations, limitations exposed by validation studies, and suggestions for modification. Kidney Int..

[10] Hu R, Xing G, Wu H, Zhang Z (2016). Clinicopathological features of idiopathic membranous nephropathy combined with IgA nephropathy: a retrospective analysis of 9 cases. Diagn. Pathol..

[11] Herlitz LC (2014). IgA nephropathy with minimal change disease. Clin. J. Am. Soc. Nephrol..

[12] Narasimhan B (2006). Characterization of kidney lesions in Indian adults: towards a renal biopsy registry. J. Nephrol..

[13] Polito MG, de Moura LA, Kirsztajn GM (2010). An overview on frequency of renal biopsy diagnosis in Brazil: clinical and pathological patterns based on 9,617 native kidney biopsies. Nephrol. Dial. Transplant..

[14] Sugiyama H (2011). Japan Renal Biopsy Registry: the first nationwide, web-based, and prospective registry system of renal biopsies in Japan. Clin. Exp. Nephrol..

[15] Chang JH (2009). Changing prevalence of glomerular diseases in Korean adults: a review of 20 years of experience. Nephrol Dial Transplant..

[16] Braden GL (2000). Changing incidence of glomerular diseases in adults. Am. J. Kidney Dis..

[17] O'Shaughnessy MM (2017). Temporal and demographic trends in glomerular disease epidemiology in the Southeastern United States, 1986–2015. Clin. J. Am. Soc. Nephrol..

[18] Hanko JB (2009). The changing pattern of adult primary glomerular disease. Nephrol. Dial. Transplant..

[19] Zhu P, Zhou FD, Wang SX, Zhao MH, Wang HY (2015). Increasing frequency of idiopathic membranous nephropathy in primary glomerular disease: a 10-year renal biopsy study from a single Chinese Nephrology Centre. Nephrology.

[20] Xu X (2016). Long-term exposure to air pollution and increased risk of membranous nephropathy in China. J. Am. Soc. Nephrol..

[21] Couser WG (2017). Primary membranous nephropathy. Clin. J. Am. Soc. Nephrol..

[22] Xu X (2016). Analysis of 4931 renal biopsy data in Central China from 1994 to 2014. Ren. Fail..

[23] Zhou Q (2018). Changes in the diagnosis of glomerular diseases in East China: a 15-year renal biopsy study. Ren. Fail..

[24] Zhou FD, Zhao MH, Zou WZ, Liu G, Wang H (2009). The changing spectrum of primary glomerular diseases within 15 years: a survey of 3331 patients in a single Chinese Centre. Nephrol. Dial. Transplant..

[25] Shin HS (2017). Patterns of renal disease in South Korea: a 20-year review of a single-center renal biopsy database. Ren. Fail..

[26] Ge YT, Liao JL, Liang W, Xiong ZY (2015). Anti-glomerular basement membrane disease combined with IgA nephropathy complicated with reversible posterior leukoencephalopathy syndrome: an unusual case. Am. J. Case Rep..

[27] Woo KT (2013). Clinicopathologic features and treatment response in nephrotic IgA nephropathy with minimal change disease. Clin. Nephrol..

[28] Mittal P (2020). Spectrum of biopsy-proven renal disease in Northern India: a single-centre study. Nephrology..

[29] Liang X (2009). Epidemiological serosurvey of hepatitis B in China—declining HBV prevalence due to hepatitis B vaccination. Vaccine..

[30] Li Y (2017). Time trends of dietary and lifestyle factors and their potential impact on diabetes burden in China. Diabetes Care.

[31] Mariachiara DC (2016). Trends in adult body-mass index in 200 countries from 1975 to 2014: a pooled analysis of 1698 population-based measurement studies with 19.2 million participants. Lancet.

[32] Nie S (2018). The spectrum of biopsy-proven glomerular diseases among children in China: a national, cross-sectional survey. Clin. J. Am. Soc. Nephrol..

[33] Hadidi R, Hadidi M, AlDabbas M (2014). Spectrum of biopsy-proven kidney disease in children at a Jordanian Hospital. Saudi J. Kidney Dis. Transplant..

[34] Printza N (2011). Percutaneous ultrasound-guided renal biopsy in children: a single centre experience. Hippokratia..

[35] Jegatheesan D (2016). Epidemiology of biopsy-proven glomerulonephritis in Queensland adults. Nephrology.

[36] Rivera F, Lopez-Gomez JM, Perez-Garcia R (2004). Clinicopathologic correlations of renal pathology in Spain. Kidney Int..

[37] Sugiyama H (2013). Japan Renal Biopsy Registry and Japan Kidney Disease Registry: committee report for 2009 and 2010. Clin. Exp. Nephrol..

