# Supplementary figures and images for: Spectrum of biopsy proven renal diseases in Central China: a 10-year retrospective study based on 34,630 cases
Source: Sci Rep. 2020 Jul 3;10:10994. doi: 10.1038/s41598-020-67910-w (PMC7335090; doi:10.1038/s41598-020-67910-w)

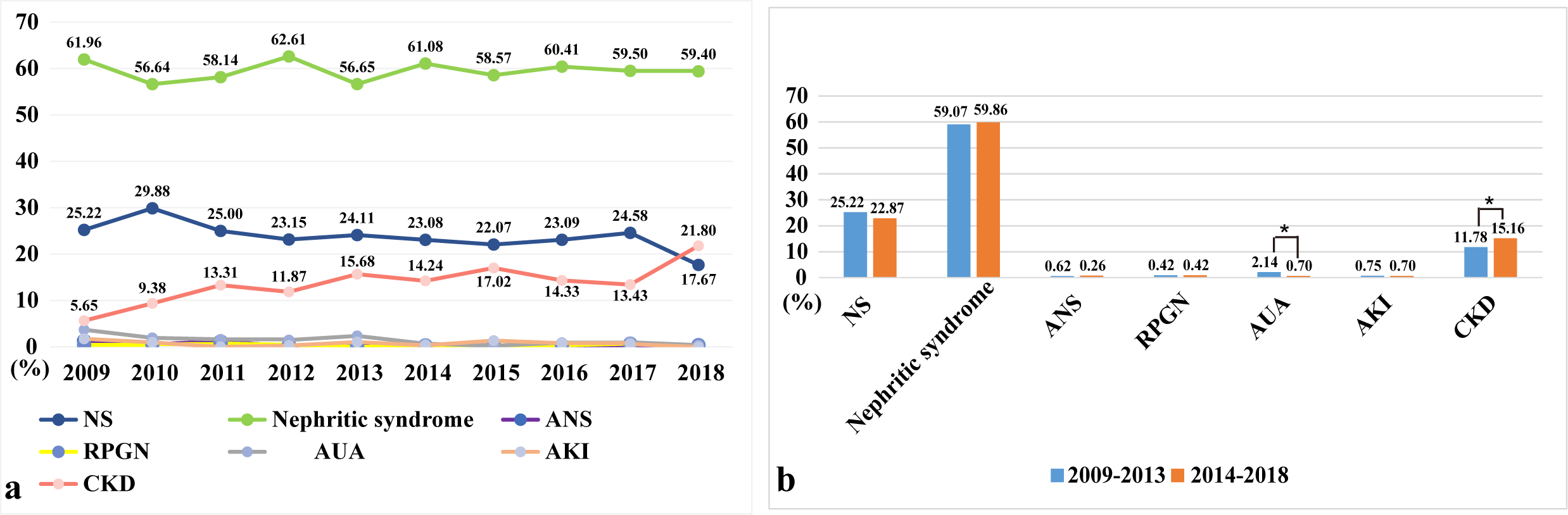

Supplement: Supplementary file 1 — Supplementary figure S1 [file 41598_2020_67910_MOESM1_ESM.tif]
