# Supplementary material for: Spectrum of biopsy proven renal diseases in Central China: a 10-year retrospective study based on 34,630 cases
Source: Sci Rep. 2020 Jul 3;10:10994. doi: 10.1038/s41598-020-67910-w (PMC7335090; doi:10.1038/s41598-020-67910-w)
Supplement: Supplementary file 2 — Supplementary information [file 41598_2020_67910_MOESM2_ESM.docx]

# Spectrum of biopsy proven renal diseases in Central China: A 10-year retrospective study based on 34,630 cases

Ruimin Hu^1^, Songxia Quan^1^, Yingzi Wang^1^, Yali Zhou^1^, Ying Zhang^1^, Lu Liu^1^, Xin J. Zhou^2*^& Guolan Xing^1*^

^1^Department of Nephrology, The First Affiliated Hospital of Zhengzhou University, Zhengzhou, Henan, People’s Republic of China

^2^Department of Pathology, Baylor University Medical Center at Dallas, Dallas, TX, USA

**Corresponding Authors:** Guolan Xing, Department of Nephrology, The First Affiliated Hospital of Zhengzhou University, Zhengzhou, Henan, People’s Republic of China,Tel: +86 0371-66913427, E-mail: [xgl@zzu.edu.cn](mailto:xgl@zzu.edu.cn)

Xin (Joseph) Zhou, Department of Pathology, Baylor University Medical Center at Dallas, Dallas, TX, USA, Tel: (972) 966-7853, Fax: (972) 966-7889, E-mail: [jzhou@pbmlabs.com](mailto:jzhou@pbmlabs.com)


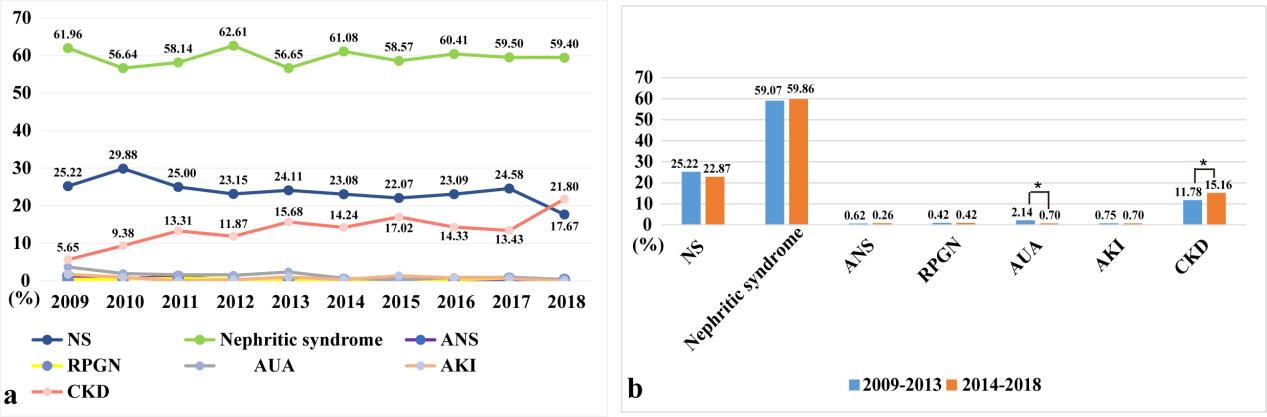


**Supplementary figure S: Annual prevalence of clinical indications for IgAN and the temporal trends.** (**a**) The annual prevalence of clinical indications for IgAN. (**b**) The changing prevalence of clinical indications for IgAN according to periods.
